# On-site analysis of cortisol in saliva based on microchannel lateral flow assay (mLFA) on polymer lab-on-a-chip (LOC)

**DOI:** 10.1007/s10544-025-00733-6

**Published:** 2025-04-10

**Authors:** V. Thiyagarajan Upaassana, Supreeth Setty, Heeyeong Jang, Sthitodhi Ghosh, Chong Ahn

**Affiliations:** Department of Electrical and Computer Engineering, University of Cincinnati, Cincinnati, Ohio 45221 USA

**Keywords:** Microchannel lateral flow assay (mLFA), Lab-on-a-chip (LOC), Unbound salivary cortisol, Competitive immunoassay, Portable fluorescence analyzer

## Abstract

Unbound cortisol in saliva, detectable through non-invasive sampling, is widely recognized as a validated biomarker for the biochemical evaluation of common mental disorders such as chronic stress, depression, anxiety, and post-traumatic stress disorder (PTSD). In this work, we report a novel polymer lab-on-a-chip (LOC) for microfluidic lateral flow assay (mLFA) with on-chip dried reagents for the detection of unbound cortisol in saliva using a competitive immunoassay protocol. The new polymer microchannel lateral flow assay on lab-on-a-chip (mLFA-LOC), replicated using injection molding technology, are composed of sequentially connected microchannels for sample loading, detection antibody immobilization, flow delay, sensing spirals for test and control, and a capillary pump at the end. The competitive immunoassay of cortisol can be autonomously performed through the microchannels after sample loading of the filtered saliva, and the fluorescence signals emitted from the sensing spirals are detected and quantified by a custom-designed, portable fluorescence analyzer developed in this work. For the evaluation of cortisol assay, artificial saliva samples spiked with unbound cortisol were analyzed using mLFA-LOC and the portable analyzer. The performed competitive assay of unbound cortisol showed a limit of detection (LoD) of 1.8 ng/mL and an inter-chip coefficient of variation (CV) of 4.0%, which covers the clinical range for on-site unbound salivary cortisol analysis. The newly developed mLFA-LOC platform certainly works successfully for the rapid on-site sampling and analysis of salivary biomarkers.

**Graphical Abstract:**

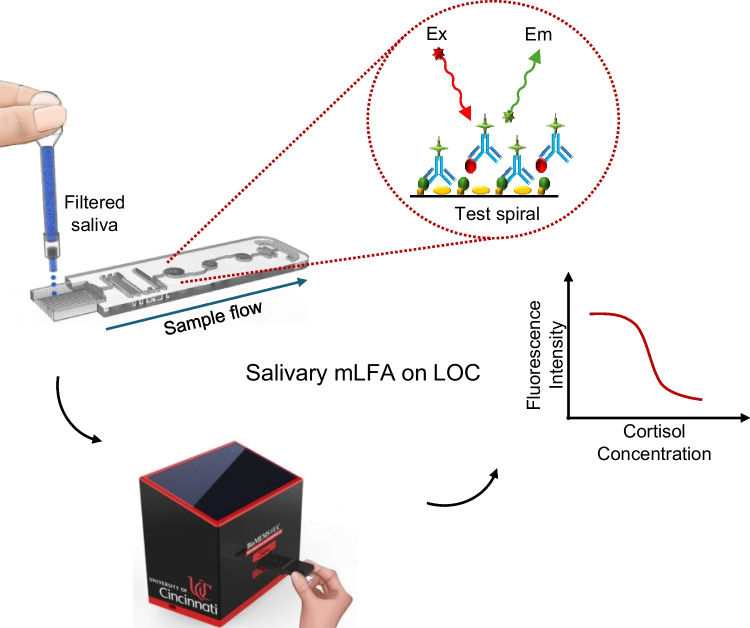

## Introduction

Detection of unbound cortisol in saliva is now considered as one of the most effective biochemical methods for the analysis of common mental disorders which affects over more than 400 million people in the world (World Health Organization 2024). Cortisol, also known as a stress hormone, is synthesized from cholesterol and acts as the main glucocorticoid in the zona fasciculate of human adrenal cortex. Its secretion in response to stress contributes to the well-characterized suppression of the hypothalamic–pituitary–adrenal (HPA) axis on health and cognition events (Hannibal and Bishop 2014). Ultimately, a prolonged or exaggerated stress response may perpetuate cortisol dysfunction, widespread inflammation, and pain (Amabebe and Anumba 2018; Kobayashi and Miyazaki 2015). In general, cortisol levels follow diurnal rhythm and cortisol levels in saliva directly reflect the amount of unbound or bioavailable hormones, unlike bounded cortisol levels in blood (Hofman 2001). Thus, continuous and rapid monitoring of cortisol levels in saliva will be a game-changer in the diagnosis of common mental disorders such as stress, depression and anxiety.

Several lab-on-a-chip (LOC)-based, microfluidic immunoassays for salivary diagnostics have been developed for point-of-care testing (POCT) applications, but most of them involve complicated and multiple steps for sample preparation, which is a critical bottleneck for the realization of POCT (Cruz et al. 2014; Kaushik et al. 2015). Electrochemical sensors (Li et al. 2023; Rahman et al. 2023) have also been developed for the POCT application of cortisol detection. In this work, we have focused on optical detection methods which uses a fluorescence detection (Zea et al. 2020).

Among several microfabrication technologies developed for the polymer LOC, the injection-molded polymer LOC with the function of capillary-driven fluid control, as our group has developed for several applications (Kim et al. 2006; Jung et al. 2011; Jung et al. 2013; Ghosh et al. 2019), is also used for the development of microfluidic lateral flow assay (mLFA)-LOC in this work. For the POCT application, the new mLFA-LOC was designed and developed as a disposable and capillary-driven (i.e., fluidic self-driven) platform with minimum human intervention. This work has explored a new fluorescence-based mLFA, using vacuum-dried reagents which are prepared inside the microchannels for POCT applications. Since cortisol is a very small molecule with a molecular weight of 362.46 g/mol, a competitive immunoassay was adopted in this work to achieve better stability compared to a sandwich immunoassay (Zainol Abidin et al. 2017).

**Fig. 1:**
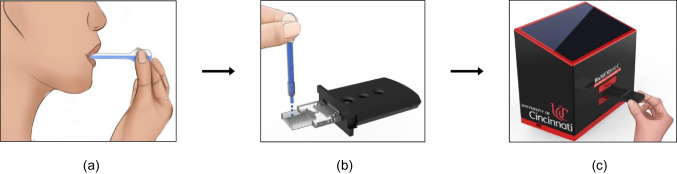
Schematic illustration of the rapid saliva sampling and on-chip immunoassay detection process: (**a**) simple and rapid saliva sampling; (**b**) dropping sample (filtered) on mLFA­-LOC; and (**c**) insertion of the closed cartridge into the portable fluorescence analyzer for quantitative POCT detection of unbound salivary cortisol

In this work, a new polymer mLFA-LOC was proposed, developed, and fully characterized as a POCT platform for the analysis of unbound cortisol in saliva samples. Most currently available saliva diagnostic kits require additional collection materials for saliva sample such as customized storage vials and separate buffer for multiple pre-treatment steps (Ghosh et al. 2019; Zainol Abidin et al. 2017). The additional sampling kit may cause the contamination or infection from mishandling the saliva samples (Rufin et al. 2015). Hence, there is a large demand for the development of an integrated or simple sampling method to address the complex issues over the saliva sampling method. We have developed a new polymer LOC with on-chip dry-reagents and simple sampling method for quantitative analysis of salivary cortisol using competitive immunoassay mechanism, which is essential for reliable and reproducible detection of very small molecule (Gubala et al. 2010). The newly developed platform combining the mLFA-LOC and the portable fluorescence analyzer was fully characterized and optimized for the optimal recovery of dried reagents and enhanced detection and quantification of unbound salivary cortisol. The demonstration of the proposed testing steps is illustrated in Fig. 1. As shown in Fig. 1, The test begins with a simple and rapid sample collection using the custom-designed sampling tool, followed by dispensing the filtered sample on the test device loaded in the cartridge and inserting the cartridge with lid-closed, in the portable fluorescence analyzer to perform the test.

## Design and fabrication of polymer mLFA-LOC

### Design of mLFA-LOC

**Fig. 2:**
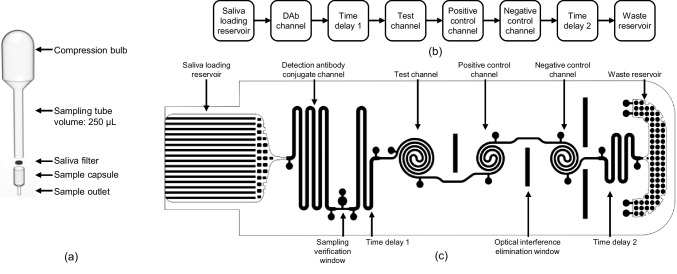
Design details of the saliva sampling tool and mLFA-LOC: (**a**) exploded schematic of the saliva sampling tool consisting of sampling tube with compression bulb for suctioning saliva directly from mouth and filtering capsule with polyurethane sponge filter and sample outlet for delivering filtered saliva on mLFA-LOC; (**b**) block diagram of the mLFA-LOC depicting the functional components; and (**c**) mLFA-LOC design with microfluidic capillary network for on-chip, dried reagents-based fluorescence immunoassay

For the collection and filtering of saliva sample directly from mouth, a simple and easy sampling tool of saliva was developed as shown in Fig. 2a. The sampling tool was composed of two parts: a sampling tube with sample container and a filter capsule with filter membrane. The developed sampling tube has a similar structure to a disposable pipette. The sampling tube could collect whole saliva when the top bulb was squeezed and then released while inserting the end of sampling tube under the tongue. As the bulb was released, the sampling tube collected around 250 μL of whole saliva pooled under the tongue. The second component, filter capsule included a filter membrane (708–20128, University Products) made of low-density polyurethane foam with a pore size of 0.3 μm. Filter membrane could remove all food particles, mucins and most importantly bubbles which could block the flow through the microchannels. After collecting saliva, sampling tube was form-fitted into the filter capsule, and approximately 50 μL of whole saliva was filtered through the outlet of filter capsule on pressing the bulb, which was directly dispensed on the saliva loading reservoir of mLFA-LOC. The saliva sampling unit was made of disposable low-density polyurethane to minimize protein binding and increase durability (Rufin et al. 2015).

To perform the competitive immunoassay for salivary cortisol detection, a new saliva mLFA-LOC was designed with a network of microfluidic capillary channels which can perform a seamless, on-chip competitive immunoassay as depicted in Fig. 2b and 2c. As shown by the block diagram in Fig. 2b, the functional mLFA-LOC components were specified as a saliva loading reservoir, a detection antibody (DAb) conjugate channel, a time delay channel for incubation time control, three spiral reaction channels for test, positive and negative control, and a waste reservoir made of capillary pump for the collection of waste fluid at the end. The design of mLFA-LOC is as shown in Fig. 2c. The sampling port (1 cm × 0.9 cm) of the mLFA-LOC was designed to hold 35 μL of filtered saliva sample. The hydrophilic microchannels in the saliva loading reservoir port of the mLFA-LOC can induce the autonomous saliva sample through a spontaneous capillary flow. Then, the saliva sample can flow through the microchannels for performing the sequential microfluidic functions desired for the competitive immunoassay.

A meander-type channel, which was located immediately after the saliva loading reservoir, was designed to contain dried-detection antibodies conjugated with a fluorescent label. Then, three spiral microchannels were followed by, for the test, positive and negative control of assay respectively, to quantitatively measure the cortisol concentration as a result of the competitive immunoassay. For comparing the assay performances of microchannels with a conventional 96-well plate, spiral microchannels were designed to have the same separation pitch to those of the wells of 96-well plate. Thus, the competitive immunoassay to be performed on the mLFA-LOC can also be read using the same microplate reader for the conventional 96-well plate, for assay comparison. Additionally, it is also beneficial to obtain comparison data between the portable analyzer and the microplate reader by comparing assay results on the mLFA-LOC, with using the data from the microplate reader as a reference (Ghosh et al. 2019).

**Fig. 3:**
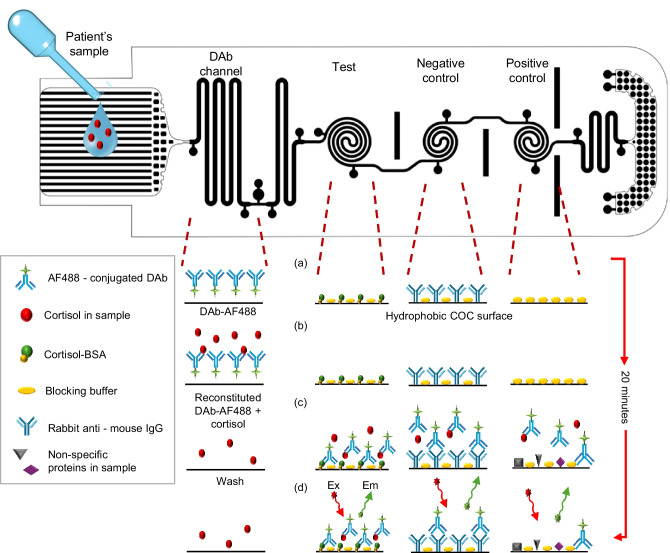
Schematic view of the developed mLFA-LOC and the sequential steps involved in performing fluorescence-based cortisol competitive immunoassay (**a**) fully prepared mLFA-LOC before sample addition; (**b**) reconstitution of DAb-AF488 in DAb channel with dispensed saliva sample containing cortisol and their binding; (**c**) remaining free DAb-AF488 binding with C-BSA in test spiral, with rabbit anti-mouse IgG in positive control spiral, and no binding in negative control spiral; and (**d**) excess loaded sample, acting as a wash buffer to remove unbound DAb-AF488 from spirals and reading the mLFA-LOC

**Fig. 4:**
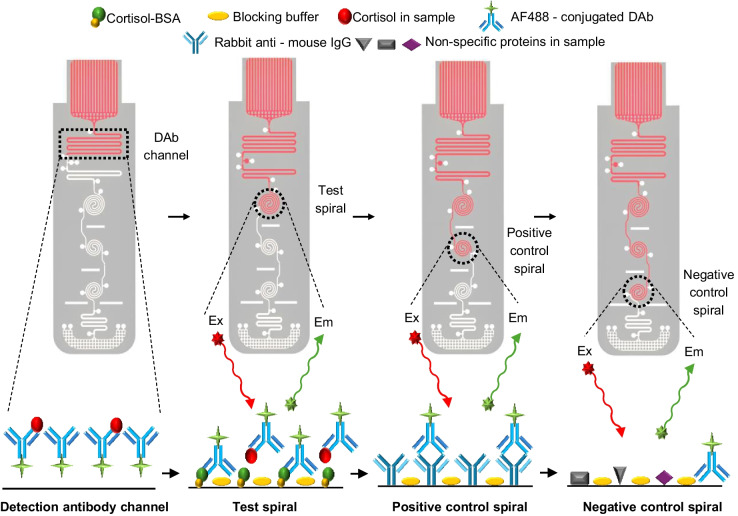
Schematic view of developed mLFA-LOC and the sequential steps involved in performing fluorescence cortisol competitive immunoassay and subsequent reading of fluorescence output signal intensity from the spiral reaction channels

A time delay channel for achieving a uniform concentration of the reconstituted DAb was added immediately after the detection antibody channel. Another delay channel positioned after the three spiral microchannels was added to slow down the sample flow, thus a longer reaction time (i.e., incubation time) between the detection antibodies and the target cortisol in the spiral channels could be attained for the improvement of assay sensitivity. At the end of channel sequence, a capillary pump-based waste chamber was placed to pull the effluent of assay which also worked as a washing buffer through the spiral channels. As the effluent was pumping into the waste chamber, the surplus sample washed the unbound detection antibodies in the spiral channels. As a result, only the bound detection antibodies in the spiral channels produced a detectable fluorescence output with a low background noise. This sequence of competitive immunoassay on mLFA-LOC is illustrated Fig. 3, where the major steps involved in the competitive immunoassay perspective. Figure 3a shows the fully prepared mLFA-LOC before the sample introduction followed by Fig. 3b, showing the reconstitution of DAb-Alexa Fluor 488 (AF488) by the patient’s sample which react among each other during a certain amount of delay before entering the sensing spirals. Figure 3c illustrates the reaction of the free DAb-AF488 with cortisol-bovine serum albumin (C-BSA) that will generate a fluorescent signal, inversely proportional to the free cortisol concentration in the sample; in positive control spiral, free DAb-AF488 will bind to the rabbit anti-mouse IgG, which will always generate a high fluorescence output utilizing for validity of the immunoassay; and the negative control shows the least output signal due to no binding, indicating non-specific binding, acting as an additional indication for the validity test. The washing step is realized by washing away unbound free DAb-AF488 by the excess sample added initially, thereby resulting in the reduced background. Figure 3d shows the immunoassay complexes formed in the sensing spiral channels after the wash step and resulting in the fluorescence signal proportional to the binding amount when excited by the filtered incident laser. Figure 4 shows the completed immuno-binding activity in each of the sensing spiral channels after the flow is complete.

To mitigate a possible optical interference between the sensing spiral channels, a small rectangular vertical cut was designed between the spiral channels which could prevent the optical interference between the channels and improve the sensitivity of the immunoassay. All dimensions and volumes for the channels and chambers of mLFA-LOC are summarized in Table 1. The saliva loading reservoir was designed to have a volume of 35 μL and the total volume of channels in the designed LOC excluding the saliva loading reservoir was 27.3 μL, slightly lower than the former so that the fluid front has enough capillarity to fill the waste reservoir.

**Table 1:** Dimensions and volumes of chambers in mLFA-LOC to facilitate dried reagent storage for fluorescence immunoassay detection of cortisol

Chambers in LOC	Width (mm)	Length (mm)	Height (mm)	Volume (μL)
Saliva loading reservoir	10	9.8	0.3	35
Detection antibody channel	0.4	68.3	0.3	8
Time delay channel 1	0.4	44.1	0.3	5.3
Test spiral channel	0.2	41.6	0.3	2.5
Positive and negative control spiral channel	0.2	25	0.3	1.5
Time delay channel 2	0.4	29.1	0.3	3.5
Waste reservior	NA	NA	0.3	5

### Fabrication of injection-molded polymer mLFA-LOC

**Fig. 5:**
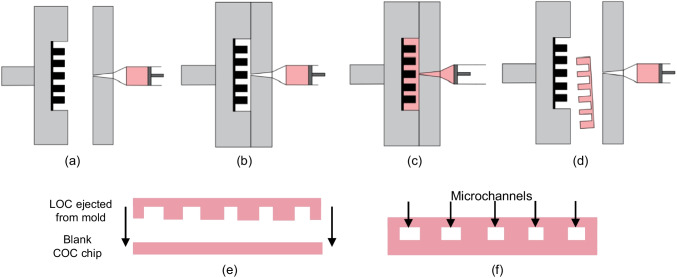
Schematic illustration of polymer mLFA-LOC fabrication process: (**a**) plasticization of COC; (**b**) mold clamping; (**c**) injection molding; (**d**) mLFA-LOC ejection; (**e**) thermal bonding at 66 kPa and 248 °F; and (**f**) thermally-bonded mLFA-LOC

**Fig. 6:**
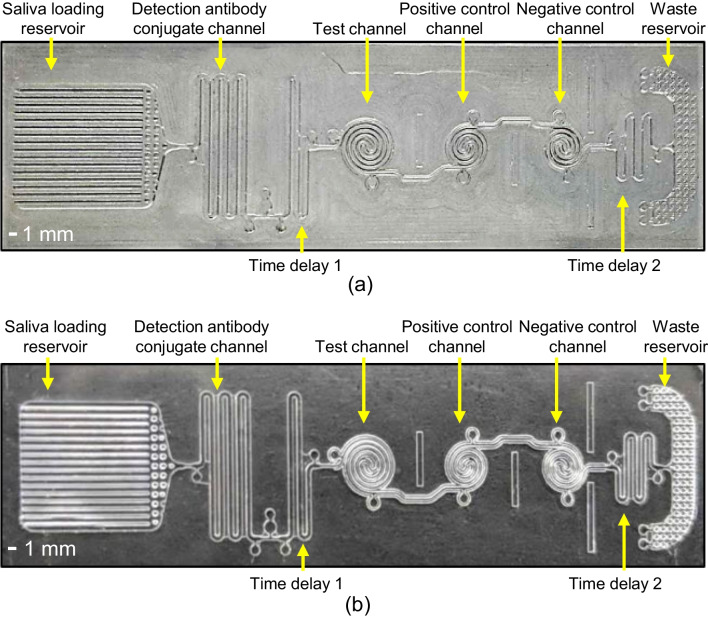
(**a**) Micromachined aluminum master mold and (**b**) replicated COC mLFA-LOC by injection molding process

Cyclic Olefin Copolymer (COC) 6013S (TOPAS Advanced Polymers) was used for the polymer material of mLFA-LOC, since COC has a flexible modification of specific or non-specific surface, excellent chemical resistance against solvents, and good optical transparency with low autofluorescence (Gubala et al. 2010; Cutroneo et al. 2023). For injection-molding of COC for the mLFA-LOC, a disk-type aluminium master mold was designed and fabricated on an aluminium metal disk using the similar method used in our previous works (Browne et al. 2011; Upaassana et al. 2019). Aluminium alloy, Alloy 6061 (McMaster-Carr, USA) was used as the material of disk-type mold which has an acceptable durability for multiple injection molding cycles. The mLFA-LOCs were injection molded using BOY 22A Procan CT (BOY Machines, USA). Injection-molded mLFA-LOCs were cut to a desired shape using CO_2_ precision laser cutting machine, VLS3.5, (Universal Laser Systems, Inc., USA) and then a blank COC plate cut to the desired shape was thermally bonded with the injection-molded mLFA-LOC to construct the microchannels at 66 kPa and 248 °F in hot press, MTP-14 (Tetrahedron Associates, USA). This fabrication process is illustrated in Fig. 5, and actual images of the fabricated master mold are shown in Fig. 6.

## Preparation of mLFA-LOC for immunoassay

### Surface modification coating of microchannel for mLFA

**Fig. 7:**
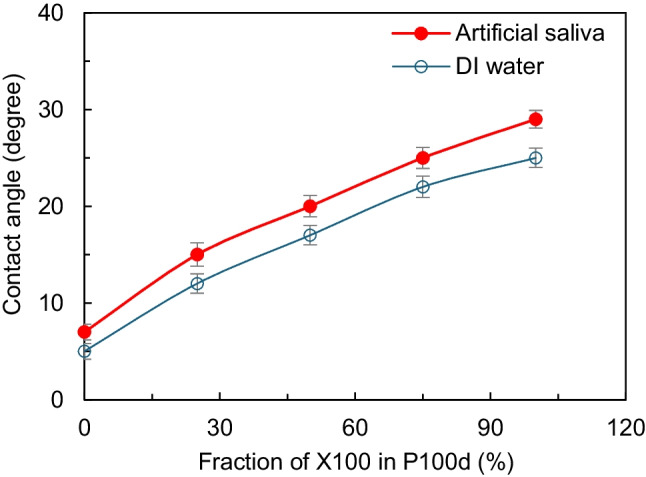
Artificial saliva and DI water contact angle analysis on a flat COC plate, dip-coated wtih different compositions of Pl00d and Xl00 hydrophilic coating solutions (Joninn, Denmark)

COC thermoplastic used for mLFA-LOC has a hydrophobic surface with a contact angle of 88°, which is not suitable for attaining sufficient capillary flow through the channels. Thus, surface modification from the hydrophobic to hydrophilic surface in the microchannel of mLFA-LOC was desired. In this work, the hydrophilic surface was modified by coating a hydrophilic coating solution P100d and X100 (Joninn, Denmark) through the channels. The hydrophilic coating solution was injected from the sampling ports to the desired microchannels with 5 minutes of incubation after filling the coating solution. The solution was pulled out and the microchannels and were vacuum dried at 100 mTorr at room temperature for 2 h in Lindberg/Blue vacuum oven, VO1218A (Thermo Fisher Scientific, USA). This hydrophilic coating allowed the microchannels to have a seamless capillary flow of saliva sample. Static contact angles for both DI water and artificial saliva (AS) were measured for the microchannels of mLFA-LOC coated with different compositions of hydrophilic coating solution. Contact angles were measured using a contact angle analyser (FTA1000 Drop Shape Instrument™, USA). As shown in Fig. 7, the measured contact angles of DI water and AS varied between 10° to 25° and 7° to 29°, respectively. These results had a similar trend to the vendor provided data (Joninn coatings datasheet. 2024).

### Reagents and materials for competitive immunoassay of cortisol

Fluorescence-based competitive immunoassay protocol of cortisol on the mLFA device is illustrated in Fig. 3. To perform the assay on the real mLFA-LOC, a couple of processes desired for the specific or non-specific surface modification and the addition of DAb-AF488 were performed. Antibody of human cortisol, ab1949 (Abcam, USA) was used as the detection antibody and it was conjugated with Alexa Fluor 488, using ab236553 (Abcam, USA) and the vendor provided protocol. Human cortisol, ab141250 (Abcam, USA) was used as the protein biomarker for spiking standards in assay buffer, Cortisol-BSA (C-BSA), 80–1434 (Fitzgerald industries, USA), EIA Diluent (Assaypro, USA) and artificial saliva for medical and dental research, 1700–0305 (Pickering Laboratories, Inc, USA). Rabbit anti-mouse IgG (RIgG), ab6709 (Abcam, USA) was used as a capture antibody in positive control spiral reaction channels, having a specific affinity in binding directly to the DAb-AF488. Reagent Diluent Concentrate 2 containing 1% bovine serum albumin (BSA) in phosphate-buffered saline (PBS) (10 mg/mL BSA in PBS), DY995 (R&D systems, USA) was used as a blocking buffer. Phosphate-buffered saline (1X PBS), DY006 (R&D systems, USA) was used as washing buffer in the Opti-96™ microplate. A benchtop 96-well plate reader, Synergy H1 (BioTek, USA), was used for reading the conventional 96-well microplate, 07–200–566, (Corning, USA), Opti-96™ microplate (Mico BioMed Co., Ltd., South Korea), and the developed mLFA-LOC.

### Vacuum-drying method for assay reagents

Vacuum-drying method, which is one of the most effective drying methods in immunology, food processing and pharmaceuticals, has been used to obtain a thin reagent coating film over the surface area of reaction chambers or channels (Bazyma et al. 2006; Davidson and Sun 2001). Vacuum-drying process can be usually performed in a low vacuum at room temperature, so it provides further simple and easy preparation of assay reagents compared to the freeze-drying (i.e., lyophilization) method. Along with vacuum pressure and temperature, the process time of vacuum-drying has a substantial effect over the quality of the dried reagent produced (Fureby et al. 1999). Hence, to obtain the desired vacuum-drying process time, 8 μL of liquid DAb-AF488 (100.0 µg/ml) was filled in hydrophilic detection antibody channel and was vacuum-dried (at 27 °C with a vacuum pressure of 100 mTorr) for varying the process time periods from 1 to 5 h, and then cortisol assay was immediately performed with the mLFA-LOC containing vacuum-dried DAb-AF488 using artificial saliva (AS) spiked with 15 ng/mL of cortisol. The process time of vacuum drying corresponding to the highest fluorescence output was chosen as the optimized time of vacuum-drying process.

Capillary flow was driven by the capillary force of microchannel surface which caused the meniscus of saliva sample pulling over the walls of hydrophilic microchannels. Since DAb-AF488 was dried on the surface of microchannel, the capillary flow velocity of sample through the microchannel determined the reconstitution efficiency of the dried DAb-AF488. To determine the optimized contact angle of AS for achieving maximum recovery of the dried DAb-AF488, fluorescence intensity exhibited from the reconstituted DAb-AF488 at three different contact angles 7°, 20° and 29° were evaluated using the fluorescence microscope, IX81 (Olympus, Inc., USA).

## Experimental results and discussion

### Cortisol assay reagent validation on conventional 96-well plate

**Fig. 8:**
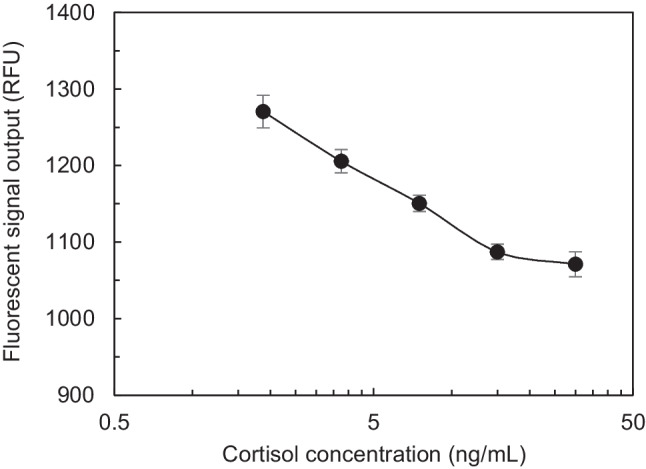
Standard curve of conventional 96-well plate competitive immunoassay for fluorescence-based detection of salivary cortisol. Results show an inversely proportional trend for the cortisol concentrations of 1.8 ng/mL to 30.0 ng/mL

As a first step towards validating assay reagent functionality, fluorescence-based cortisol assay was performed first on a conventional 96-well plate using competitive assay protocol as follows. 100.0 μL of C-BSA (50.0 µg/ml) was incubated overnight in black conventional 96-well microplate and after a wash, 100.0 μL of blocking buffer was incubated for 2 h. After washing again, 100.0 μL equal mixture of different standard solutions and DAb-AF488 (25.0 μg/mL) were incubated for 2 h. After a final wash process, the plate was read with an excitation wavelength of 485 nm and emission wavelength of 525 nm. As shown in Fig. 8, fluorescence output signals decreased with increasing cortisol concentrations from 1.8 ng/mL to 30.0 ng/mL (two-fold dilution in assay buffer). Since competitive fluorescence assay involves competition between standard cortisol and C-BSA, fluorescence output signal was observed to be linearly decreasing as cortisol concentration increased.

### Antibody optimization for microchannel based assay

In the microchannel-based assay using mLFA-LOC, protein binding kinetics depends on the rate of diffusion of the reagents, which is influenced by flow rate and the concentration of the reagents. In order to maximize the chance to capture the antigen in flowing microchannel-based immunoassay, a high surface concentration of the immobilized capture antibodies increases the number of antigen binding to the antibodies, which ensures the high fluorescence signal output. Thus, an assay optimization is required for finding the optimal C-BSA and DAb-AF488 concentrations compatible with the application of mLFA-LOC. The competitive assay of cortisol was performed by widely varying the concentration of DAb-AF488 and C-BSA to be optimized while the concentrations of all other assay reagents and the assay protocol had remained fixed.

The assay performances in spiral microchannels of mLFA-LOC needed to be compared with those on conventional 96-well plate, so a microchannel-based assay platform such as Opti-96™ microchannel microplate (Mico BioMed Co., Ltd., South Korea), which was recently developed and announced (Kai et al. 2012; Ahn et al. 2011), was utilized. Spiral microchannels of Opti-96™ microplate was developed to have the same diameter of reaction unit and separation pitch to that of 96-well plate. Thus, the assay performance of Opti-96™ microchannel microplate can be directly compared with 96-well plate without changing microplate reader for signal detection. Similarly, the spiral channels of mLFA-LOC were designed to have the same dimension and separation pitch to both 96-well plate and Opti-96™ microplate. Thus, the assay performances of mLFA-LOC can be progressively evaluated and compared with those of 96-well plate first and then Opti-96™ microplate as a reference or standard.

For the assay on Opti-96™ microplate, a checkerboard assay format proceeded with increasing the capture protein concentration horizontally and increasing the detection antibody concentration vertically across the Opti-96™ microplate. Based on the obtained assay results, the antibody concentration exhibiting the highest fluorescence signal was determined to be the optimal concentration for all further assays (referring the Assay Transfer Guide, Mico BioMed) (Opti-96™ assay transfer guide 2024).

**Fig. 9:**
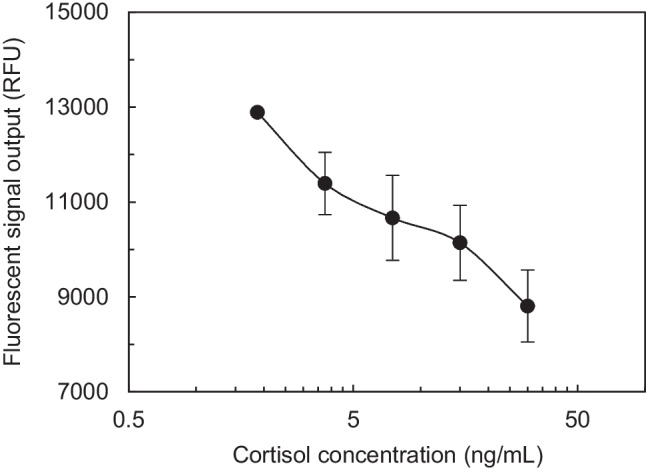
Assay results obtained from Opti-96™ microplate assay for fluorescence-based detection of salivary cortisol (1.8 ng/mL to 30.0 ng/mL). Results show an inversely proportional trend, like conventional 96-well plate results

All cortisol antibody optimization assays on Opti-96™ microplate were performed based on the following protocol: (a) 5.0 μL of C-BSA was incubated for 20 minutes, followed by blocking for 10 minutes using 5.0 μL of blocking buffer; (b) 5.0 μL equal mixture of sample containing cortisol and DAb-AF488 was incubated for 10 minutes followed by a washing step using 5.0 μL of PBS; (c) then, the plate was read after 5 minutes with an excitation wavelength of 485 nm and emission wavelength of 525 nm. Table 2 shows the summary of the performed checkerboard assay results. The highest fluorescence signal was achieved with a DAb-AF488 concentration of 200.0 µg/ml (8X) and C-BSA(2X) concentration of 100.0 µg/ml. Followed by C-BSA and DAb-AF488 optimization for cortisol antigen detection, optimal concentration of RIgG was determined to be used in mLFA-LOC. Immunoassay protocol used for RIgG optimization was very similar to the cortisol antibody optimization, without cortisol incubation step. The highest output fluorescence signal was observed for 0.1 mg/ml which was fixed to be optimized value for all further assays. Although the antibody concentrations optimized for the microfluidic platform was higher than those of the conventional 96-well plate assays, the volume of reagents used on Opti-96™ microplate was 20 times smaller which provided cost-effectiveness for POCT applications. In the final step after optimizing the antibody concentration, cortisol immunoassay (spiked artificial saliva samples) was performed on Opti-96™ microplate using optimized C-BSA and DAb-AF488 concentration. Assay results obtained from Opti-96™ microplate is shown in Fig. 9. It can be observed that the fluorescence output signal provided a higher resolution for the same cortisol standard detection range, 0.93 ng/mL to 0.93 ng/mL, when compared to conventional 96-well plate results (Fig. 8). However, the signals obtained from Opti-96™ microplate showed larger values than those of 96-well plate, which is a general outcome observed due to the higher surface-to-volume ratio of the microchannels. It also might be due to the variation of process conditions that needed to be further optimized. Since the vacuum-dried process of DAb-AF488 required for the microchannel based assay, it was successfully optimised using the spiral microchannel of Opti-96™ microplate, the evaluation of competitive assay on mLFA-LOC was ready to move to the preparation of mLFA-LOC using a similar optimization protocol developed in this section.

**Table 2:** Cortisol antibody and Cortisol-BSA optimization checker-board assay obtained from Opti-96™ microplate. Fluorescence assay signal variation with the increase C-BSA concentration and DAb-AF488 concentration at a fixed antigen concentration (30.0 ng/mL)

Cheker-board optimization		Cortisol-BSA							
		2x				4x			
		Antigen 30.0 ng/mL	C.V.(%)	Negative control	C.V.(%)	Antigen 30.0 ng/mL	C.V.(%)	Negative control	C.V.(%)
Cortisol antibody	2x	6855.5	3.1	6145	0.3	7094.5	0.4	6105	0.7
	4x	8178.5	5.0	6243.5	1.7	8326	3.2	6436.5	2.6
	8x	11,606.5	2.8	7109	1.1	10,391.5	4.6	9031	4.9

### Process of vacuum-drying reagents for competitive assay on mLFA-LOC

Artificial saliva samples spiked with unbound cortisol will be dispensed on the saliva loading reservoir using a squeeze-drop filler as illustrated in Fig. 1, then the sample flows sequentially through the channels without losing the target cortisol to the spiral channels and successfully reconstituting the vacuum-dried reagents. Thus, three major processes desired for the surface modification and reagent addition on the mLFA-LOC are (a) Optimization of blocking buffer required to prevent non-specific binding on the surface as well as achieving desired contact angle for seamless flow of sample liquid through the microchannels; (b) Optimization of DAb-AF488 vacuum-dried process required for the DAb and bound with the vacuum-dried DAb-AF488 forming DAb-AF488-cortisol complex which eventually reached the spiral reaction test channel containing immobilized and blocked C-BSA; and (c) Optimization of hydrophilicity for the delay channel, achieving a uniformly reconstituted reagent flow and optimized incubation in the spiral channels.

#### Optimization of blocking buffer

As reported in our previous works, the fundamental principle of protein adsorption on the COC surface is through hydrophobic interactions with a low energy surface and there is very poor protein adsorption on hydrophilic COC surfaces with high surface energy (Waghmare and Mitra 2013). BSA coated polymer surfaces have been reported to exhibit hydrophilic nature (Bazyma et al. 2006; Bialopiotrowicz and Janczuk 2001; Yu et al. 2016), and hence hydrophilicity of spiral reaction channels for sample flow was controlled using a blocking buffer with 1% BSA in PBS. For the characterization of the contact angle formed by BSA adsorption, plain COC chips (3 cm X 3 cm X 1.1 mm) were dip-coated with 1% BSA in PBS for different periods ranging from 5 to 45 minutes and vacuum-dried for 2 h at 100 mTorr to ensure complete drying. The contact angles of DI water and AS measured on BSA coated COC chips were applied to the spiral reaction channels to control the capillary flow of sample.

**Fig. 10:**
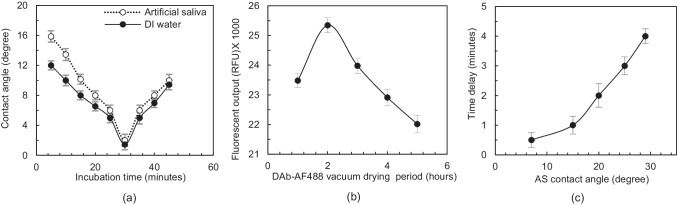
(**a**) Contact angle analysis on flat COC plate, dip-coated in 1% BSA in PBS with varying incubation times. Contact angle decreases as incubation time increases up to 30 minutes, followed by which contact angle increases gradually when the incubation time is increased from 30 to 45 minutes; (**b**) fluorescence-based lateral flow immunoassay results for detection of cortisol on LOC, vacuum-dried for varying periods of time from 1 to 5 h. 15 ng/ml of cortisol spiked with artificial saliva was used as sample; and (**c**) sample flow delay obtained in the time delay chambers of mLFA­ LOC coated with five different AS contact angles, 7°, 15° 20°, 25° and 29°. Time delay increased with increasing the contact angle

As the results shown in Fig. 10a, AS contact angle decreased with increasing incubation time with the lowest contact angle (1 ~ 2°) at 30 minutes, followed by which there was a gradual increase in the AS contact angle as the incubation period increased from 30 to 45 minutes. The initial decrease in the AS contact angle as the BSA concentration was increased was due to a monolayer coverage of BSA molecules with free polar groups. This monolayer facilitated the wetting of polar liquids like AS and saliva, but the AS contact angle increased after 30 minutes of incubation due to the formation of double layer of BSA causing reduction in surface energy available for adhesion (Lee and Ruckenstein 1988). Based on the results observed, optimized incubation time was chosen to be 30 minutes which allowed a highest capillary flow in the spiral reaction channels.

#### Optimization of DAb-AF488 vacuum drying process

As shown in Fig. 10b, a highest fluorescence intensity from the spiral reaction channels was obtained from mLFA-LOC which were vacuum-dried for 2 h, however a gradual decline of fluorescence intensity was observed in the devices which were dried for further longer hours. The decrease in fluorescence signal output indicated the adverse effects on functionality of DAb-AF488 due to the agglomeration and denaturation of antibodies (Sou et al. 2015; Mumenthaler et al. 1994). Since microfluidic capillary flow is laminar, flow velocity determined the reconstitution efficiency of vacuum-dried DAb-AF488 which in turn depended on the AS contact angle in microchannel.

### Delay time control using contact angle

To ensure a longer incubation time between cortisol antibodies and target cortisol for improving the assay performance, flowing times of sample through the time delay channels with five different AS contact angle, 7°, 15°, 20°, 25°, and 29° were explored and a contact angle corresponding to the longest time delay was chosen.

As shown in Fig. 10c, the highest time delay of 4 minutes was obtained from the contact angle of 29° which was due to the lowest hydrophilicity. Thus, both time delay channels were coated with X100 hydrophilic coating (AS contact angle ~ 29°), to ensure a minimum 4 minutes of incubation time between the cortisol antibodies and the free cortisol in dispensed saliva sample. The final step of assay process was washing using a surplus saliva sample, so the waste reservoir was designed and fabricated to have an AS contact angle of 29° which achieved a slow movement of surplus sample and thus efficiently removed the unbound antibodies.

In this stage, all microchannels were properly coated to seamlessly flow the saliva sample, and the preparation of assay reagents as a dried form inside the microchannels and spirals was done on the mLFA-LOC. Thus, the mLFA-LOC was fully ready to perform the cortisol assay by just dispensing the filtered saliva sample over the sampling port. In order to use the mLFA-LOC as a POCT platform, we also designed and developed a custom designed portable fluorescence analyzer, which is described in the following section.

### Development of a portable fluorescence analyzer

**Fig. 11:**
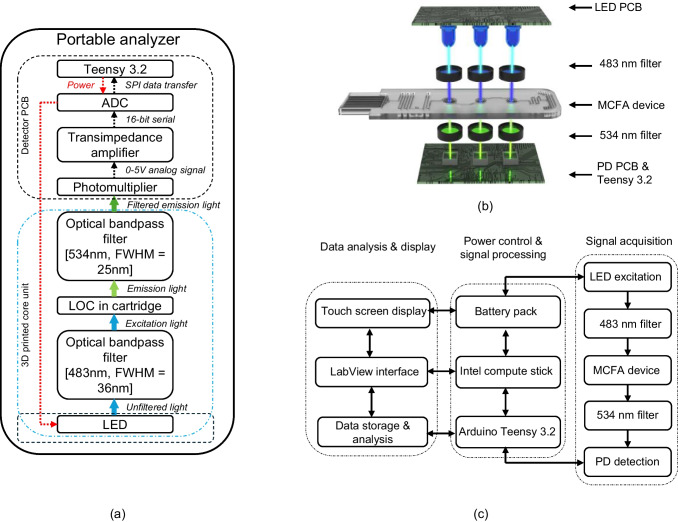
(**a**) Block diagram of the portable fluorescence analyzer depicting the electronic and optical modules along with the flow of measured fluorescent signals from the mLFA-LOC; (**b**) schematic of transmission-based fluorescence intensity measurements detected using mLFA-LOC in portable analyzer; and (**c**) block diagram of the portable fluorescent analyzer mechanism for quantitative detection of salivary cortisol using mLFA-LOC

Major disadvantages for the current benchtop analyzer are bulkiness and high cost which is unsuitable for point-of-care applications and low-resource settings (Lakowicz 1988; Shahzad et al. 2009). Hence, a portable fluorescence analyzer was designed to fit the developed mLFA device for point-of-care detection of salivary cortisol. COC substrate has 90% optical clarity with high transparency and very low autofluorescence which make it compatible for transmission-based fluorescence intensity measurements. Compared to reflection-based fluorescence intensity measurements, transmission-fluorescence setup does not require a dichroic mirror or extra real estate for angling emitted light towards the photodetector. Hence, spiral reaction channels of mLFA device were sandwiched in between excitation source and photodetector with appropriate filters for selectively filtering peak excitation wavelength and peak emission wavelength. A 3D-enclosure setup was optimally designed to efficiently direct excitation light towards mLFA-LOC and emission light towards photodetector with minimum external interference and transmission losses. A block diagram depicting the components, and the flow of measured fluorescence signal is shown in Fig. 11a.

### Electrical and optical system design

As depicted in Fig. 11b, the basic principle of fluorescence analyzer is to excite the fluorophore labels (AF-488 in this case) conjugated to DAb with 490 nm light emitting diode (LED) and detect the filtered emission light (525 nm) using high-sensitive silicon photodiode (PD). The mLFA-LOC was sandwiched between excitation LED and emission sensing PD with respective filters (483 nm—#67–014, Edmund optics; 534 nm #18–391, Edmund optics, USA). LED490L (Thor labs, Inc, USA) was used as excitation source and silicon photomultipliers, C series (SensL Technologies, Ltd., USA) were used as photodetectors. The exploded view of this setup is illustrated in Fig. 11b. Teensy 3.2, DEV-13736 (Sparkfun electronics, USA) and Intel® Compute stick, STK1AW32SC (32 GB, Windows 10, Intel®, USA) were used to drive LED circuit and PD circuit along with communication for data processing. Intel compute stick is a powerful single-board computer on a flash drive which supports graphic programming software in plug-n-play format with HDMI port connections. 5″ capacitive touch screen, 800 × 480 HDMI monitor TFT LCD (GeekPi, Amazon, USA) was used as a display screen to read fluorescence intensity and a rechargeable 5 V battery pack, A1216 (13,400 mAh, Anker technology, China) was used to power the system and fully charged system provided a standalone run-time of 9 hours under continuous use. The complete simplified block diagram of the portable fluorescence analyzer is split into signal acquisition, power control and signal processing, and data analysis and display units as shown in Fig. 11c. Sensing components used in portable fluorescence analyzer were explicitly chosen to fit the spiral dimensions of mLFA device. This eliminated potential loss of output optical signal from out-of-focus detection region.

### Analyzer 3D setup and mLFA-LOC cartridge

**Fig. 12:**
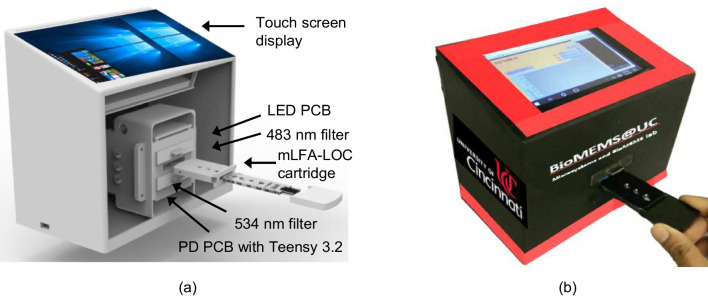
(**a**) 3D enclosure parts of the fluorescence analyzer: different layers of electrical components stacked to sandwich mLFA-LOC in between excitation LED source and detection photomultiplier using corresponding optical filters for AF 488 along with 3D printed cartridge containing apertures for fluorescence excitation and detection of spirals; and (**b**) fully assembled portable fluorescence analyzer with mLFA-LOC cartridge (enclosing saliva chip) for point-of-care quantitative detection of salivary cortisol

Geometrical and mechanical alignment of spiral reaction channels in mLFA-LOC with the LEDs, PDs and filters are of utmost and critical importance to achieve accurate, reliable and repeatable output. Since high-sensitive PDs are responsive to small changes of light intensities from excitation source and ambience, robust and leak-free electrical housing and enclosures were designed to minimize external electromagnetic interference without compromising user comfort and compactness. As shown in Fig. 12a, 3D printed stack was designed to house LED circuit board, 483 nm filters, 534 nm filters and PD circuit board in vertical stack on top of each other with a center slot for cartridge with mLFA-LOC. This setup created a vertical stack of LED/483 nm filter/cartridge with mLFA-LOC/534 nm filter/PD which facilitated transmission-based fluorescence detection. A separate housing on top of the core stack held Intel compute stick, battery pack and touch screen display with accommodations for running wired connections between all the electrical components. To shield mLFA-LOC from external contamination, the cartridge was designed with a removable cap. Cartridge body was designed with three apertures on top and bottom walls which aligned with the spirals to allow access for excitation and fluorescence emission intensity measurements. After dispensing the sample, cartridge cap is closed and inserted into the analyzer with the long end facing the cartridge slot, such that cartridge cap remained outside the analyzer and shielded sampling reservoir. A mechanical click-lock mechanism was employed to eliminate movement of cartridge inside the enclosure housing while performing fluorescence intensity measurements. The fully assembled fluorescence analyzer as shown in Fig. 12b had dimensions of 6 cm × 11 cm × 15 cm and was easily transportable for performing measurements in POC settings with a long battery life. Thus, upon saliva sample loading, the cortisol assay was autonomously performed on the mLFA-LOC and analyzed using the developed portable fluorescence analyzer by simply inserting the cartridge of mLFA-LOC into the analyzer’s slot.

### Assay results performed on mLFA-LOC

**Fig. 13:**
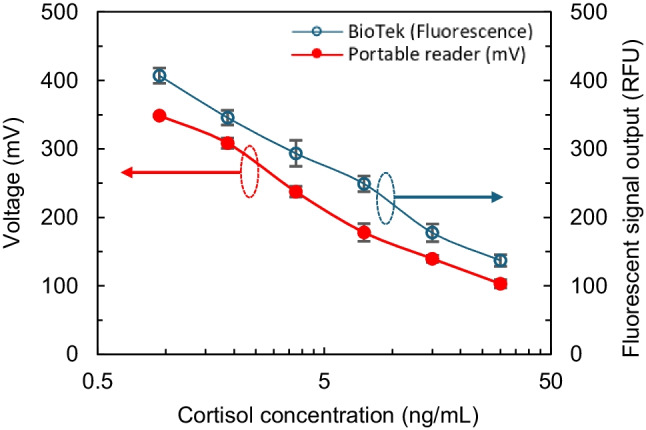
Salivary cortisol assay results obtained with spiked artificial saliva on mLFA-LOC, measured using the portable fluorescence analyzer for concentrations ranging from 0.93 ng/mL to 30.0 ng/mL (red plot). The same mLFA-LOCs are further read using the gold standard BioTek reader (blue plot)

The mLFA-LOC developed in this work with on-chip dried immunoassay reagents were tested with spiked AS samples which contained different concentrations of cortisol to evaluate the performance of the mLFA-LOC. Upon dispensing sample to the saliva loading reservoir, the sample started to flow through the microfluidic channels due to capillary flow with no external pressure or flow control. The volume of microchannel was designed to have a desired sample volume to fill the final spiral reaction channels. Thus, once the required microfluidic capillary flow steps were completed, the flowing sample allowed the measurements of fluorescence intensity for a range of cortisol concentrations. From the observation of the flow through the mLFA-LOC, it was found to take 20 minutes for the sample to flow from the sample pad to the end of waste reservoir. Then, at 20 minutes after loading a sample, the closed cartridge with the mLFA device was inserted into the portable fluorescence analyzer. The assay results obtained from the mLFA-LOC, which were measured from the developed portable fluorescence analyzer, are shown in Fig. 13, indicated by the red plot. The results obtained from both the custom-designed portable fluorescence analyzer and the gold standard benchtop BioTek reader show a similar trend for the variation of cortisol concentrations with a wide detection range and high sensitivity. It was observed that there was a linear decrease in fluorescence output signal with increase in cortisol concentration from 0.93 ng/mL to 30.0 ng/mL with an inter-chip Coefficient of Variation (C.V.) of less than 4.0%. The choice of this detection range is because this range covers the cortisol levels that normally vary in humans throughout the day and in case of disorders that affect cortisol levels and vice versa and fall in the clinically accepted range (Pearlmutter et al. 2020; Laudat et al. 1988; Ahn et al. 2007; Zangheri et al. 2015). The linear trend of the graph is also very similar to the standard curve obtained from the 96-well plate and the Opti-96™ plate shown in Figs. 8 and 9 respectively. The LoD obtained for cortisol detection with the developed mLFA-LOC and fluorescence analyzer was 1.8 ng/mL.

## Conclusion

In this work, for the first time, we have successfully developed a new lab-on-a-chip for the microchannel lateral flow assay (mLFA), which can autonomously perform a rapid and high-sensitive immunoassay for detecting unbound salivary cortisol, using a competitive assay protocol. The developed mLFA-LOC worked successfully with the capillary-driven flow mechanism with on-chip dried immunoassay reagents, which had successfully performed the fluorescence-based competitive immunoassay for a quantitative analysis for unbound salivary cortisol. The achieved LoD and detection range using mLFA-LOC for the cortisol were measured around 1.8 ng/mL and 0.93 ng/mL – 30.0 ng/mL, respectively. In conclusion, the mLFA-LOC developed in this work has envisaged as a new POCT platform for on-site cortisol test using non-invasive samples and can be applied as a POCT platform for numerous on-site clinical diagnostics using saliva, urine, or plasma samples.

## Data Availability

No datasets were generated or analysed during the current study.
